# Sitosterol and glucosylceramide cooperative transversal and lateral uneven distribution in plant membranes

**DOI:** 10.1038/s41598-021-00696-7

**Published:** 2021-11-03

**Authors:** V. Rondelli, A. Koutsioubas, J. Pršić, E. Deboever, J. M. Crowet, L. Lins, M. Deleu

**Affiliations:** 1 Department of Medical Biotechnology and Translational Medicine, Università degli Studi di Milano, Milano, Italy; 2 Jülich Centre for Neutron Science at Heinz Maier-Leibnitz Zentrum, Forschungszentrum Jülich GmbH, Garching, Germany; 3 Microbial Processes and Interactions Laboratory (MiPI), TERRA Research Center, Gembloux Agro-Bio Tech, Université de Liège, Gembloux, Belgium; 4 Laboratoire de Biophysique Moléculaire aux Interfaces, Structure Fédérative de Recherche Condorcet, TERRA Research Center, Gembloux Agro-Bio Tech, Université de Liège, Gembloux, Belgium; 5 Laboratory of Natural Molecules Chemistry, Gembloux Agro-Bio Tech, University of Liège, 2, Passage des Déportés, 5030 Gembloux, Belgium; 6 FytoFend S.A., rue Georges Legrand, 6, 5032 Isnes, Belgium; 7 Université de Reims Champagne-Ardenne, UFR Sciences Exactes et Naturelles, Reims, France

**Keywords:** Computational biophysics, Membrane biophysics, Membrane structure and assembly, Biomimetics, Plant biotechnology, Biophysics, Biotechnology, Computational biology and bioinformatics, Plant sciences, Plant molecular biology

## Abstract

The properties of biomembranes depend on the presence, local structure and relative distribution assumed by the thousands of components it is made of. As for animal cells, plant membranes have been demonstrated to be organized in subdomains with different persistence lengths and times. In plant cells, sitosterol has been demonstrated to confer to phospholipid membranes a more ordered structure while among lipids, glycosphingolipids are claimed to form rafts where they tightly pack with sterols. Glucosylceramides are glycosphingolipids involved in plant signalling and are essential for viability of cells and whole plant. The glucosylceramide-sitosterol structural coupling within PLPC membranes is here investigated by Langmuir films, in silico simulations and neutron reflectometry, unveiling that a strong direct interaction between the two molecules exists and governs their lateral and transversal distribution within membrane leaflets. The understanding of the driving forces governing specific molecules clustering and segregation in subdomains, such as glucosylceramide and sitosterol, have an impact on the mechanical properties of biomembranes and could reflect in the other membrane molecules partitioning and activity.

## Introduction

Plasma membrane is a complex functional object, whose different activities depend on the presence of local structure assumed by the thousands of components it is made of. Not only the presence of specific molecules plays a role for membrane functions, but also their localization within the membrane. As for animal cells, plant membranes have been demonstrated to be organized in subdomains with different persistence lengths and times^1–4^. Both the lateral and cross distributions of proteins and lipids concur in finely tuning the structure and functionality of membrane domains and their ability to respond to external stimuli. Lipids, essential for membrane structuring, are also key molecules for cell physiology. Within the membrane, they can laterally segregate and self-assemble in micro- and nanodomains, promoting protein aggregation and the formation of molecular functional platforms^5^. An example are the so called ’rafts’, membrane domains with a low degree of fluidity, typically enriched in sterols and sphingolipids, whose structure changes in space and time as a response to external events. They form dynamic signalling complexes generating cascade events to regulate cellular processes, eventually, but not forcely, involving proteins^6–11^. In plant cells, sterols enrich the plasma membrane^12^. While animal and fungal membranes contain one major sterol class each, cholesterol and ergosterol, respectively, plant membranes contain a variety of sterols, such as stigmasterol, sitosterol and campesterol for the most abundant^13^. Plant sitosterol has been demonstrated to confer to phospholipid membranes a more ordered structure, similarly to the well characterized cholesterol in animal membranes^14–16^. Among plant sphingolipids, glucosylceramides (GluCer), consisting of a glucose head-group bound to a ceramide backbone, are involved in signalling and are essential for viability of cells and whole plant^17,18^. Molecular dynamics simulations^19^ showed that in a dilinoleoylphosphocholine (DLPC) membrane, clusters of GluCer and sitosterol form, indicating the tendency of these molecules to aggregate in domains separated from unsaturated phospholipids. Moreover, it has been shown that model membranes mimicking plant plasma membranes are less temperature sensitive than those of animals, suggesting that components like sitosterol and glucosylcerebrosides are produced in order to extend the temperature range in which membrane-associated biological processes can take place^2^.

Not only the lateral distribution of components is crucial for membrane structure and functionality, but also their distribution in the two leaflets facing the inner cell space or the outer environment. Membrane asymmetry is a key feature for cells. While for animal cells, it is well characterized, it was not widely experimentally addressed for plant membrane^20–22^. Asymmetry quantification is an hard task to achieve, but for *Avena sativa*, it has been suggested that 65% of phospholipids are found in the inner leaflet, while 70% of the total content of sterols and GluCer reside in the outer one^23^. The quantification of asymmetry in membrane domains is an even harder goal to be achieved, mainly because of the difficulty to isolate rafts with detergents without generating artefacts, or without the use of membrane-intercalating dyes impacting membrane properties. However, it is of great importance since the understanding of their organization will contribute to deciphering their role in cell physiology^24^. The investigation of different kinds of plant membrane structures have been profitably achieved by the application of radiation scattering techniques on natural and model systems, in particular to investigate mitochondrial membranes and to unveil the effect of sterols on membrane structure^25–31^.

In this context, the understanding of the driving forces governing the direct or indirect aggregation and segregation of specific molecules, such as GluCer and sitosterol, in subdomains, needs further investigation^32^. Specific molecular couplings, indeed, besides impacting the mechanical properties of the membrane portions in which they reside, could modulate the partitioning of other molecules, such as proteins or other lipids, found within the same membrane portion.

To unveil these aspects, we focused on the sitosterol-GluCer pair and we investigated their structural coupling within palmitoyl-2-linoleoyl-sn-glycero-3-phosphocholine (PLPC) membrane by complementary biophysical techniques. Surface pressure-area Langmuir curves on pure and mixed lipid monolayers aimed at investigating specific interactions among components in the mixture; molecular dynamics simulations shed light on the tendency of the three molecules to partition laterally within the membrane; neutron reflectometry helped in quantifying the spontaneous distribution of components within the two leaflets of single supported membranes. Our results indicate that a direct interaction between GluCer and sitosterol exists and governs their lateral and transversal distribution within PLPC membrane leaflets.

## Results and discussion

### Lipid packing: langmuir films at the air/water interface

Surface pressure (Π) − molecular area (A) compression isotherms were recorded to measure the surface pressure after the single components and their mixture were separately spread on a Tris HCl subphase at pH 7.4 and 25 °C. Analysis of the compression isotherms of mixed monolayer gives information about the mutual lateral packing of its components. Within a mixed monolayer, if the two components are immiscible (or ideally miscible), the area occupied by the mixed film will be the sum of the areas of the separated components (obeying the additivity rule^33^). Any deviation from the additivity rule can be attributed to a specific interaction between the components^33,34^.

**Figure 1:**
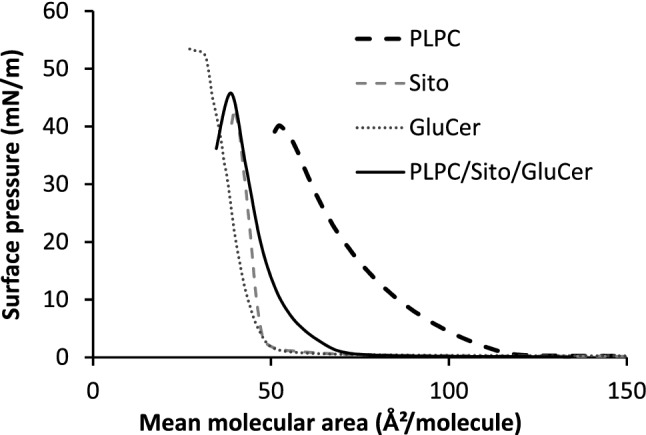
Surface pressure–area (Π–A) compression isotherms, at the air–water interface, of monolayers of pure PLPC, sitosterol (Sito), and glucosylceramide (GluCer), and of their ternary mixture (60/20/20 molar ratio) recorded at 25 °C with a Tris HCl subphase at pH 7.4. Triplicate experiments using independent preparations yielded similar results.

In Fig. 1, the compression isotherm of the ternary mixture PLPC/sitosterol/GluCer (60/20/20 molar ratio) is compared to the ones of the pure lipid components. At a surface pressure of 30 mN/m, presumed to be the one prevailing within biological membranes^35^, the mean molecular area of the mixed monolayer with a plant plasma membrane biomimetic composition (PLPC/sitosterol/GluCer, 60/20/20 molar ratio) (43.4 ± 0.7 Å^2^/molecule), is 10 Å^2^ less than the theoretical value (A_123_ = 53.2 ± 1.0 Å^2^) calculated from the additivity rule based on the mean molecular of pure components (see material and methods). It suggests a specific attractive interaction between the components^33,36,37^ giving rise to a condensing effect of their organization at the air–water interface.

Sterols like cholesterol are known to induce the condensation of acyl chains of low-melting lipids. A template mechanism in which the sterol planar nucleus maximizes its contact with the flexible acyl chains in order to produce a large number of close hydrophobic contacts and tight packing, is strongly preferred against the umbrella hypothesis in which acyl chains and cholesterol become more tightly packed because they share limited space under phospholipid headgroups^38,39^. In presence of low-melting kinked phospholipids, cholesterol is also suggested to be pushed away from these lipids and pulled towards high-melting ones like sphingolipids^39^. This push–pull mechanism is proposed to be an active mechanism for the formation of lipid rafts enriched in sterol and sphingolipids.

To get further insight into the lateral organization of our plant membrane models, we first used molecular dynamics simulations.

### Lipid lateral organization: computer simulations

**Figure 2:**
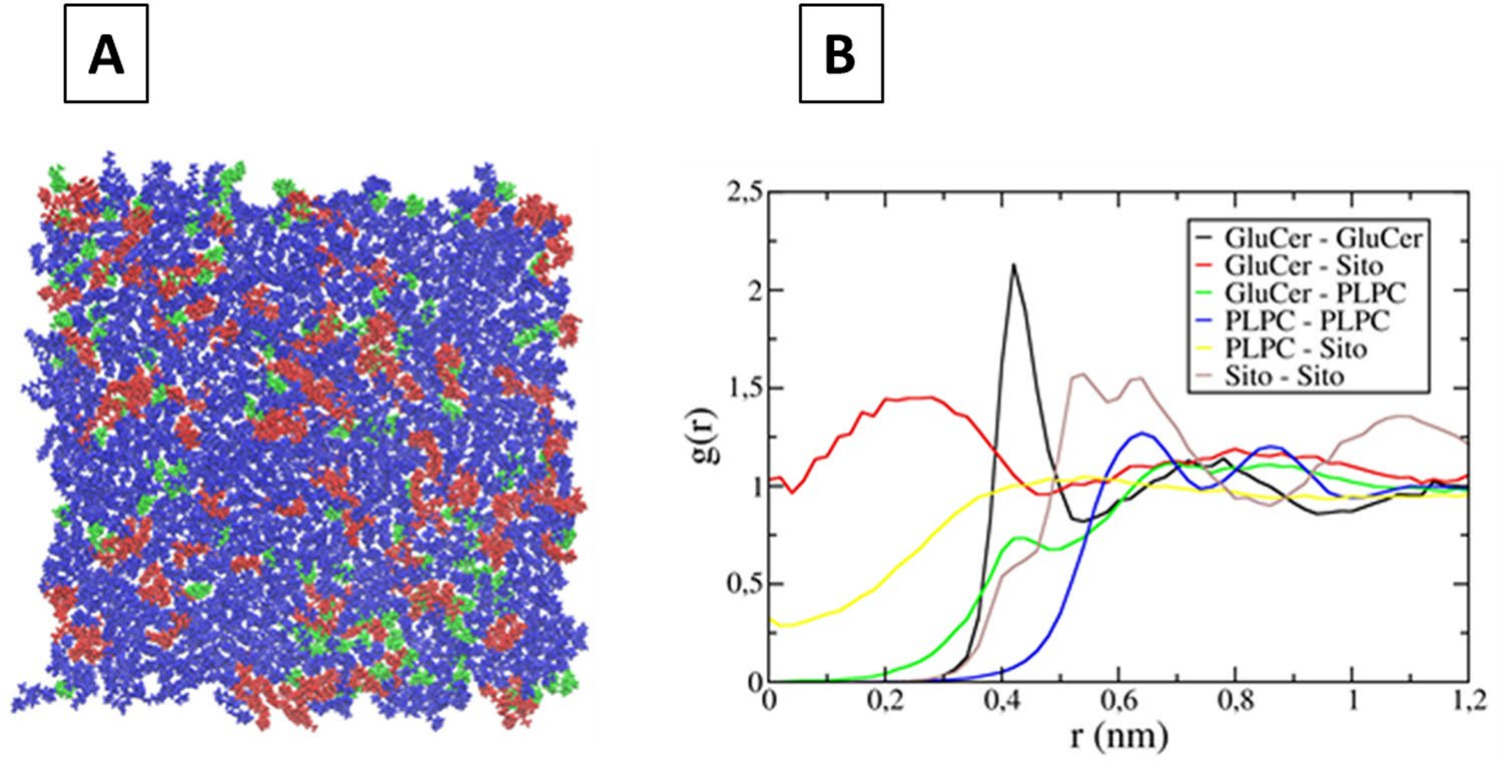
Molecular dynamics simulations on PLPC/sitosterol/GluCer (60/20/20) bilayer. Blue: PLPC, green: sitosterol (Sito) and red: GluCer. (**A**) Top view of the bilayer at the end of the simulation; (**B**) radial distribution of the different lipids in the membrane plane. It represents the probability density (g(r)) to find a lipid species (the second name of the legend) at a certain distance (r) of a defined lipid species (the first name of the legend) along the trajectory.

Molecular dynamics simulations performed on PLPC/sitosterol/GluCer (60/20/20) bilayer show clearly the formation of GluCer/sitosterol clusters within a PLPC matrix (Fig. 2A). According to the radial distribution data, sitosterol is preferentially localized within these clusters (Fig. 2B).

Our results show that half of the GluCer are in interaction with other GluCer and that around 40% of the sitosterol molecules are in interaction with GluCer (see Figures S1 and S2).

Our simulations are in agreement with those of Emami et al.^19^ showing a clustering of GluCer and sitosterol when the molar ratio of GluCer is equal or higher than 15.5%, and is thus not in favour of the hypothesis reported by Varela et al*.*^40^ saying that GluCer has a low tendency to associate with sterol-enriched domains.

**Figure 3:**
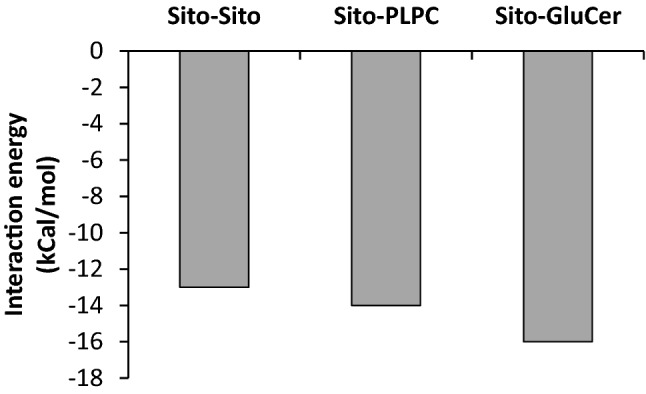
Interaction energy (Kcal/mol) between lipid molecules calculated from docking calculations (Hypermatrix).

The preferential interaction between GluCer and sitosterol is confirmed by the interaction energy calculation using the docking method Hypermatrix (Fig. 3) showing a higher negative interaction energy for the sitosterol-GluCer couple than for sitosterol-sitosterol and sitosterol-PLPC.

### Cross-sectional structure: neutron reflectometry

#### PLPC/sitosterol/GluCer

After silicon characterization and membrane deposition, we measured reflectometry from the plant model membrane composed by PLPC/sitosterol/GluCer in 60/20/20 molar proportion. Reflectometry has been collected from the system at room temperature in three contrast solvents: H_2_O, D_2_O and a mixture of the two with contrast 4*10^-6^ Å^−2^ (4-match water, 4 MW).

Worthnoting in the present experiments, for the first time a single membrane containing both sterols and glycosphingolipids has been successfully deposited by the easy to handle technique of fusion of vesicles and has been characterized by neutron reflection.

An initial data analysis was performed using a model-free approach^41^. The estimated overall layer thickness (D_max) was found to be equal to 65 Å. D_max also includes the native ≈10 Å silicon oxide layer on the substrate. The simulated annealing minimization led to an estimation of solvent volume fraction and SLD profiles near the silicon surface (see figures in SI). The general characteristic silicon oxide (≈10 Å) / thin water layer (≈5 Å) / lipid head (≈10 Å) / lipid tail (≈30 Å) / lipid head (≈10 Å) structure is reflected in the SLD profiles.

**Figure 4:**
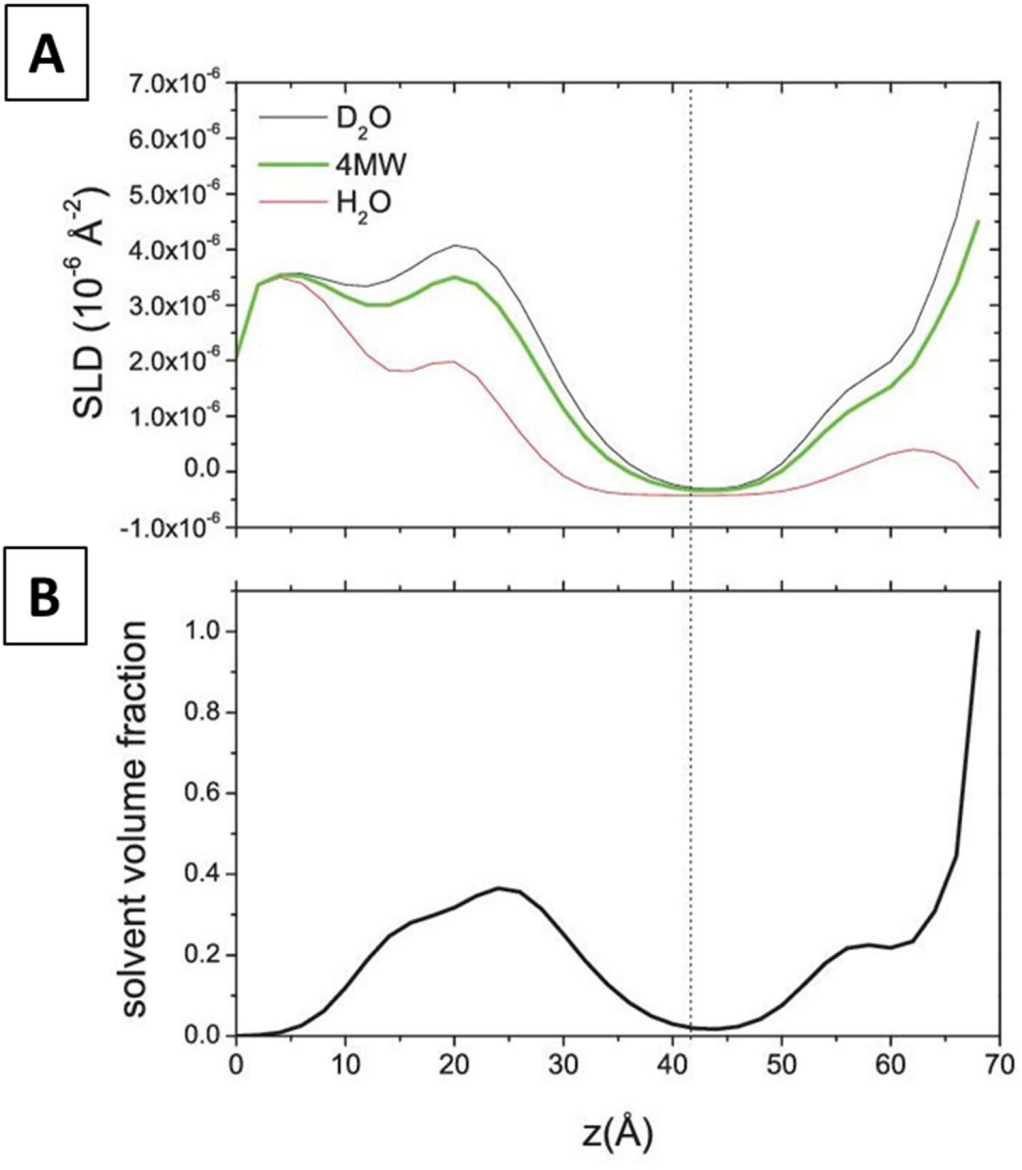
(**A**) SLD profiles and (**B**) solvent volume fraction for PLPC/sitosterol/GluCer (60/20/20) membrane obtained with the model-free approach. Dotted line represents the center between upper and lower leaflet headgroup region.

By a closer inspection of the obtained profiles, in all PLPC/sitosterol/GluCer bilayer measurements, an asymmetry between the lower and upper leaflet is evident, that registers both as a relatively higher solvent content and SLD of the membrane leaflet closer to the substrate (Fig. 4).

**Figure 5:**
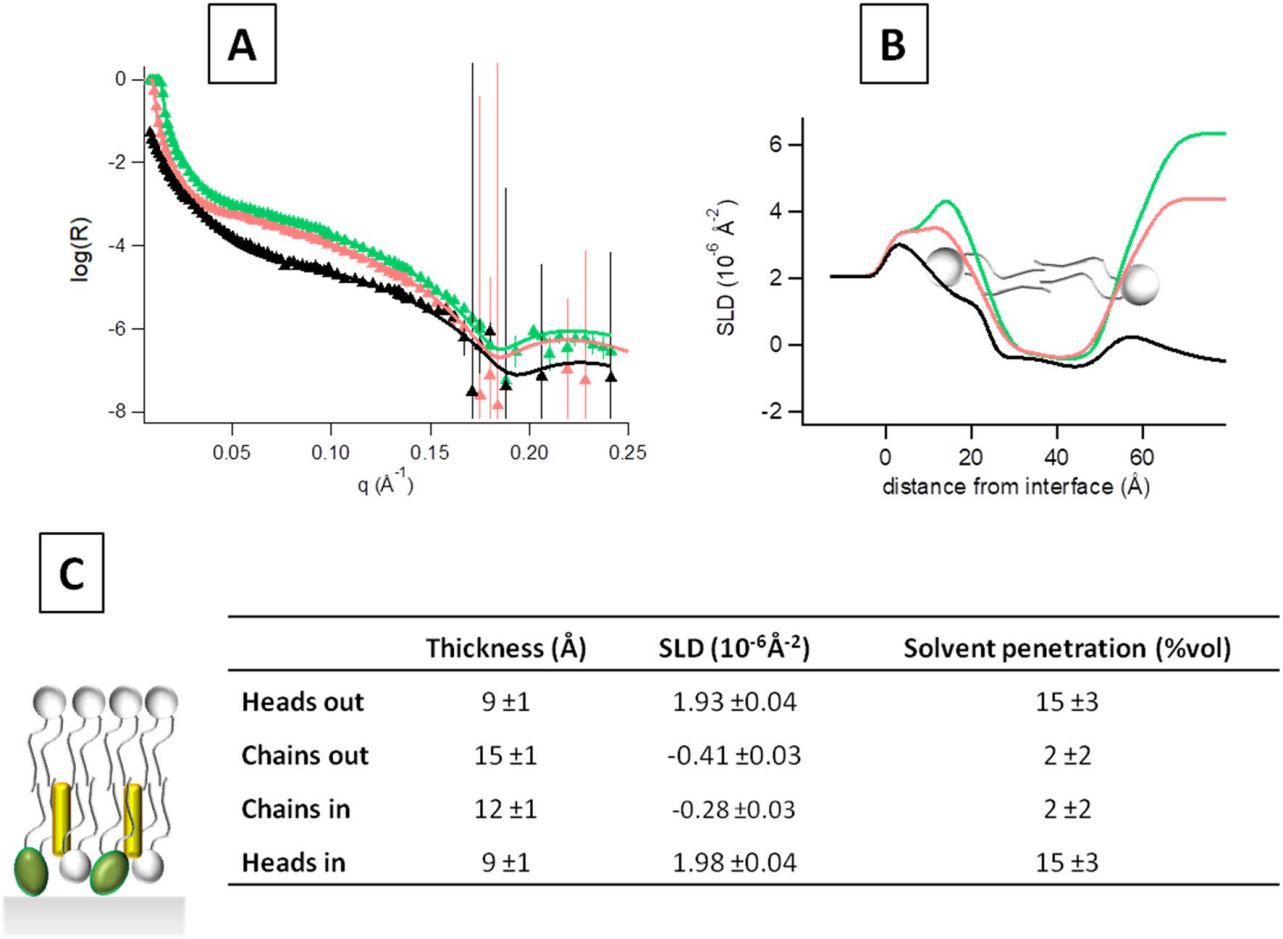
(**A**) Reflectivity curves of the supported PLPC/sitosterol/GluCer (60/20/20) membrane investigated in D_2_O (green), H_2_O (black) and 4 MW (pink) at room temperature. Symbols are the experimental point, lines the best fits, from which the SLD profiles showed in panel (**B**) are obtained with the software MOTOFIT^42,43^. (**C**) Common structural parameters used to contemporary fit the curves relative to the PLPC/sitosterol/GluCer membrane in the three contrast solvents. Errors on the single values have been determined from the fit quality though the χ^2^ value.

Given the complexity of the system under study, the model-free analysis provides un-biased information about the actual SLD profiles, that may be complemented by a model-based data refinement using MOTOFIT software^42,43^ version for Igor 6, giving further insight concerning the structural parameters of the different layers. Data, best fits and derived SLD profiles are represented in Fig. 5A, B, while the common parameters used for contemporary fit are reported in Fig. 5C.

Data analysis indicates that the deposited membrane coverage was almost perfect although the presence of glycolipids, with a packing parameter P < 1, in the membrane and generally speaking the presence of several components may affect the coverage rate^44,45^.

The interlayer roughness was found to be low, between 2 and 3 Å and a 4 Å thick water layer between the membrane and the silicon block was detected. As in the model-free approach, the membrane is found to be asymmetric. In the model-free approach, where no assumptions are made about the layer structure, a hint about the asymmetry is obtained, and is reflected both in the layer SLD and solvent penetration. The model-based analysis with MOTOFIT^42,43^, possibly more realistic, indicates instead that the asymmetry is mainly due to differences in the SLD and not in the two leaflets hydration.

In particular, data analysis reveals that, in the planar system formed after fusion, sitosterol assumes an asymmetric distribution between the two leaflets. Actually, it was found to reside into the inner leaflet of the membrane (in proximity of silicon), where the contrast reaches a value of − 0.28*10^−6^ Å^−2^ significantly higher than that of the bare lipid chains (− 0.41*10^−6^ Å^−2^), being the contrast of sitosterol 0.27*10^-6^ Å^−2^.

This uneven distribution of sterols in ceramide-based lipid membranes appears to be in good agreement with previous results on GM1 and cholesterol-containing lipid membranes^45^. The difference between the two systems is quantitative because while in presence of GM1 cholesterol assumes a partial asymmetry (80% is found in the layer opposite to that of GM1, while the remaining 20% reside in the same membrane leaflet of GM1), here the asymmetry is total: all the sitosterol is found to reside within the inner layer of the plant mimic membrane.

Unfortunately, GluCer distribution through the membrane cannot be quantified, but only guessed, since its contrast with respect to PLPC is too poor and only originated from the polar heads moiety (2.2*10^−6^ Å^−2^ versus 1.93 * 10^−6^ Å^−2^ of PLPC heads). Nonetheless, a higher solvent penetration content was detected in the proximal membrane leaflet (see Fig. 4), the one facing the silicon block, where sitosterol was found to reside. This lower coverage rate, detected as higher leaflet ‘solvent penetration’ may indicate that GluCer resides as well on that membrane leaflet, together with sitosterol. Indeed, glycosphingolipids, bearing highly hydrated and voluminous polar portions, possibly contribute to the enhancement of the hydration of the membrane leaflet they reside in.

This hypothesis, supported by the literature^23^, brings us to assume that GluCer co-localizes with sitosterol within the inner leaflet of the supported membrane. This finding is different from what was already found for the couple cholesterol-GM1, for which the glycolipid demonstrated to localize in the layer opposite to that containing the majority of the sterols^45^.

Independent measurements on several ternary lipid mixtures have been performed, giving the same results, reported in the SI.

#### PLPC/sitosterol

To check whether the asymmetry we highlighted was deriving from some specific interaction with the silicon support as already described in the literature^46,47^ or truly due to the coupling between sitosterol and GluCer, as already found for cholesterol and GM1 ganglioside^45,48^, we investigated the same system in a model membrane lacking glycolipids.

As before, after silicon characterization and membrane deposition we measured reflectometry from the bicomponent membrane PLPC/sitosterol 80/20 mol in two contrast solvents: H_2_O and D_2_O at room temperature.

**Figure 6:**
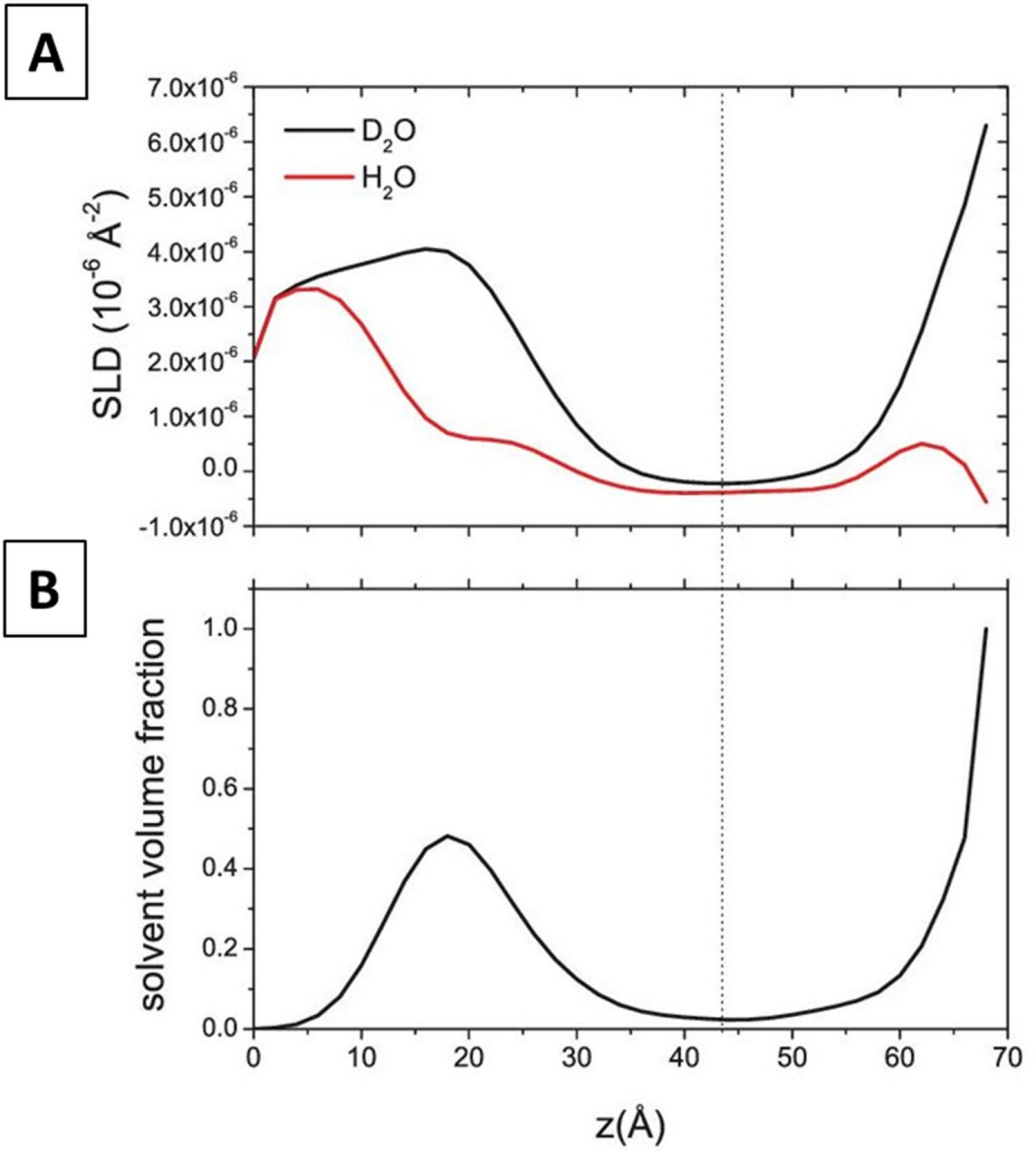
(**A**) SLD profiles and (**B**) solvent volume fraction for PLPC/sitosterol (80/20) membrane obtained with the model-free approach. Dotted line represents the center between upper and lower leaflet headgroup region.

Again, an initial data analysis was performed using a model-free approach^41^ and the estimated overall layer thickness (D_max) was found to be equal to 65 Å in this case as well. The simulated annealing minimization led also here to an estimation of solvent volume fraction and SLD profiles near the silicon surface (see figures in SI). By a closer inspection of the obtained profiles, PLPC/sitosterol bilayer shows a high surface coverage and a symmetric membrane structure (Fig. 6), contrarily to what was observed for the ternary mixture.

**Figure 7:**
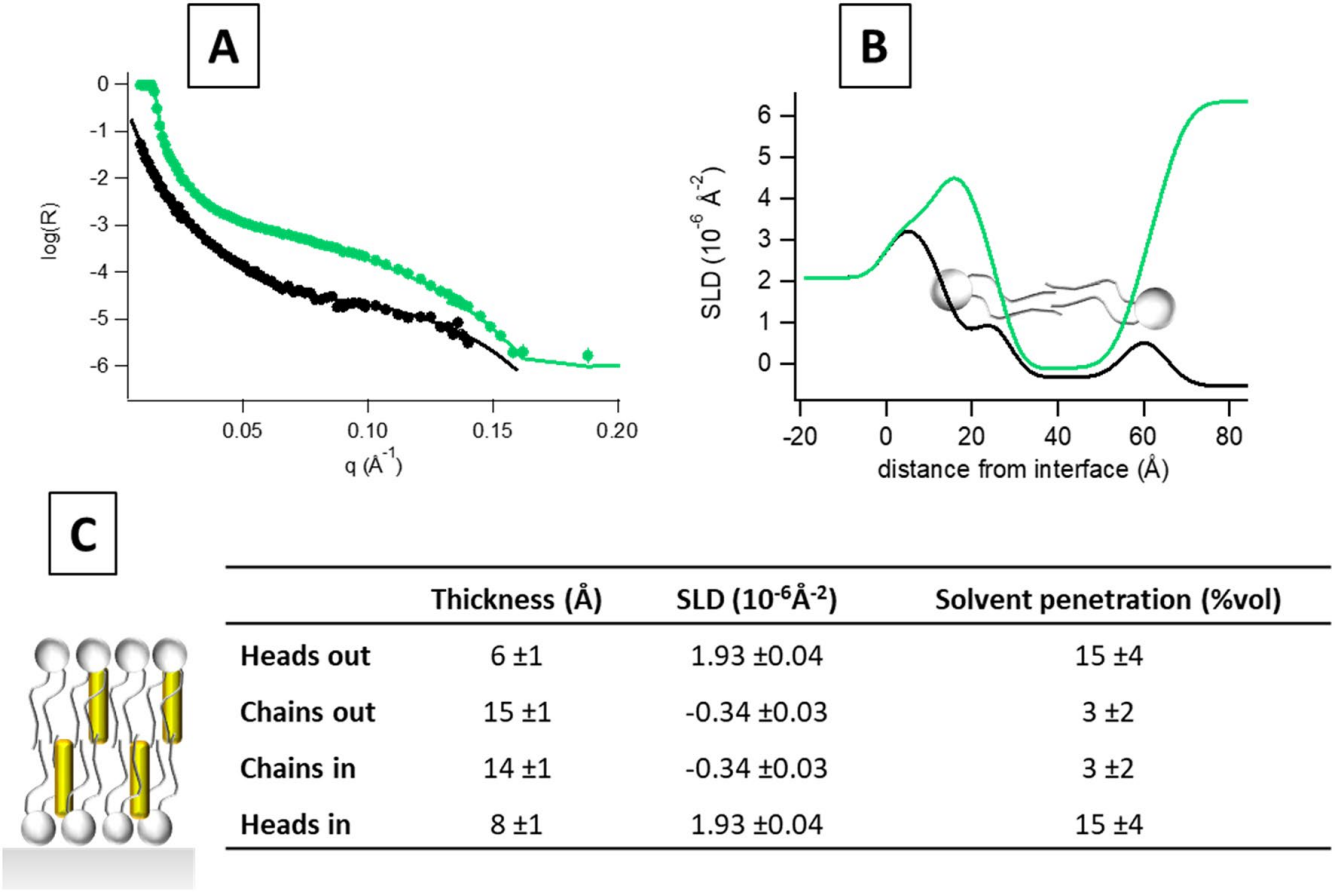
(**A**) reflectivity curves of the supported PLPC/sitosterol (80/20) membrane investigated in D_2_O (green) and H_2_O (black) at room temperature are shown. Symbols are the experimental points, lines the best fits from which the SLD profiles shown in panel (**B**) have been obtained with the software MOTOFIT^42,43^. (**C**) Common structural parameters used to contemporary fit the curves relative to the PLPC/sitosterol (80/20) in H_2_O and D_2_O at room temperature. Errors on the single values have been determined from the fit quality though the χ^2^ value.

A more detailed insight of the layer structure was obtained by data analysis with software MOTOFIT^42,43^. Best fits, derived SLD profiles and the fitting parameters obtained are reported in Fig. 7.

Data analysis indicates that membrane coverage was very high (97%), the interlayer roughness was found to be between 3 and 4 Å and a 6 Å thick water layer between the membrane and the silicon block was found.

In this system, a full membrane symmetry was found. Sterol symmetry in phospholipid membranes is not surprising, as already described with cholesterol^45,49^.

This result supports the hypothesis that sitosterol asymmetry observed in the ternary mixture is due to the presence of GluCer.

Our results as a whole suggest that a structural coupling between sitosterol and GluCer occurs, and is reflected in the two molecules lateral and transverse distribution within the PLPC matrix. A similar strong coupling was already demonstrated to exist between the two plasma membrane components cholesterol and GM1 ganglioside^48^.

The coupling of the raft-forming sterols and glycosphingolipids, as sitosterol and GluCer, is of wide interest, being potentially related to lipid raft, and therefore plant membrane functionality.

## Conclusion

Assessing the cross and lateral distribution of the molecules within cell membrane is a difficult task, especially with non-invasive techniques, but it is of fundamental interest for the understanding, and eventual manipulation, of cell functionality at the molecular level.

Here we exploited in silico and experimental techniques to investigate the sitosterol-GluCer coupling within PLPC membranes, enriching plant membrane rafts.

The complementary biophysical approaches used allowed to demonstrate that a strong direct interaction between sitosterol and GluCer exists within plant model membranes and governs their lateral and cross distribution in membrane leaflets. Indeed, sitosterol and GluCer are find to laterally co-segregate within the PLPC matrix, and to co-localize in the same membrane leaflet, finally found to be fully asymmetric concerning their distribution.

The importance of this finding is enriched by the fact that none of the used technique is invasive for the system such as the use of fluorophores or other ionizing radiations may be, even if our approach required the use of simplified systems. However, part of our studies have been performed on monolayer systems, while the presence of an opposite layer could affect the components lateral distribution, as in real membranes the presence of other molecular components or transient situations occurring at membrane surface may affect molecular distributions. Future investigation will lead to study more complex membrane models, where other molecules, as proteins, will be embedded, in order to unveil the processes governing cross and lateral components distribution within the membrane at the molecular level, reflecting in membrane structural and dynamical properties.

## Materials and methods

### Materials

Palmitoyl-2-linoleoyl-sn-glycero-3-phosphocholine (PLPC), sitosterol and d-glucosyl-ß-1,1′-*N*-palmitoyl-d-erythro-sphingosine (d18:1/16:0) (Glucosylceramide or GluCer) were purchased from Avanti Polar Lipids and used without further purification.

### Vesicles preparation

PLPC/sitosterol 80/20 mol and PLPC/sitosterol/GlcCer 60/20/20 mol mixed system have been obtained by mixing the correct amounts of each component dissolved in organic solvent, then dry films have been formed on a ball-shaped glass flask, finally hydrated to the 0.5 mg/mL concentration in NaCl 150 mM water and extruded through filters with a porosity of 80 nm to obtain monolamellar vesicles.

### Vesicles deposition for single membrane preparation

After silicon characterization, supported bilayers have been formed by the vesicle fusion technique^43^: vesicles of the desired composition have been injected in the NR measuring cell at room temperature. After 45 min for incubation and bilayer deposition the excess vesicles have been removed by gentle solvent exchange.

### Langmuir films

Surface pressure (Π) − molecular area (A) compression isotherms were recorded using an automated LB system (KSV Minitrough [width,75 mm; area, 24.225 mm^2^], KSV Instruments, Helsinki, Finland) with a platinum Wilhelmy plate to measure the surface pressure. Lipids (PLPC, sitosterol, glucosylceramide) were dissolved in chloroform/methanol (2:1, v/v). Pure solutions as well as their ternary mixture (PLPC/sitosterol/GluCer (60/20/20)) were prepared to a final concentration of 1 mM. A volume of 20–30 μL was spread on the Tris HCl subphase at pH 7.4 and 25 °C. After waiting for 15 min for solvent evaporation and spreading of the molecules, the monolayer was compressed by two barriers approaching symmetrically at a rate of 10 mm/min. The surface pressure was measured during the entire compression. The variation coefficient of at least two independent experiments did not exceed 5%.

The additivity rule was applied according to the following equation to calculate the theoretical value of the mean lipid molecular area in the ternary mixture PLPC/sitosterol/GluCer (60/20/20):

$${\text{A}}_{123} = {\text{X}}_{1} {\text{A}}_{1} + {\text{X}}_{2} {\text{A}}_{2} + {\text{X}}_{3} {\text{A}}_{3}$$

where X is the molar fraction and the subscripts 1, 2, 3 and 123 refer to the pure components 1, 2 and 3, and to their ternary mixture, respectively.

### Molecular dynamics

PLPC/sitosterol 80/20 mol and PLPC/sitosterol/GluCer 60/20/20 mol mixed system (1020 lipids) have been studied by molecular dynamics (MD). All simulations have been performed with the CHARMM36 force field^50^. Membrane systems have been generated by using the CHARMM-GUI membrane builder^51–54^ and the box filled with TIP3P water^55^ with a water/lipid ratio of 50 and a KCl concentration of 150 mM. All the systems studied were equilibrated by using the six steps equilibration proposed by the CHARMM-GUI membrane builder; a minimization by steepest descent of 1,000 steps, two NVT and four NPT simulations with increasing length and time step and decreasing restraints force constants on lipids phosphate positions and dihedrals. Temperature and pressure were coupled at 295 K and 1 bar respectively using the weak coupling Berendsen algorithm with τT = 1 ps and τP = 5 ps^56^. Pressure was coupled semiisotropically. The production simulations were then carried out for 1000 ns and as triplicate for the PLPC/sitosterol/GluCer system and for 50 ns for the PLPC/Sitosterol system. Periodic boundary conditions (PBC) are used with a 2 fs time step. Temperature was maintained by using the Nosé-Hoover method^57,58^ and pressure by using the Parrinello-Rahman barostat^59^ with a compressibility of 4.5 × 10^5 (1/bar). Electrostatic interactions were treated by using the particle mesh Ewald (PME) method^60^. The van der Waals interaction was switched off from 1 to 1.2 nm by the force-based switching function^61^. Hydrogen bonds lengths were maintained with the LINCS algorithm^62^. Trajectories were performed and analyzed with GROMACS 2020.3 tools^63^. 3D structures were analyzed with both PYMOL (Schrödinger2010) and VMD^64^ softwares. Radial distribution functions have been computed for the last 400 ns of the trajectories with the PLPC phosphate, ceramide first carbon and sitosterol oxygen. A value of 1 means a uniform distribution. At distance lower than 0.3 nm, values tend to 0 because lipids cannot interpenetrate except for sterols with GluCer and to a lesser extent with PLPC, meaning that sterols are located beneath the first lipid species.

### Hypermatrix

Hypermatrix is a simple docking method described in^65,66^. Like it was recently done for saponins^67^ and amphiphilic azobenzenes^68^, it was used to calculate the paired interaction between sitosterol and PLPC or GluCer. Sitosterol is fixed at a central position of the system and oriented at the hydrophobic/hydrophilic interface and the interacting molecule (PLPC or GluCer), also oriented at the lipid/water interface, is positioned around the central molecule by rotations and translations (more than 10^7^ positions tested). For each position, an interaction energy is calculated, based on an empirical forcefield^69^. The most stable assembly is the one having the lowest energy value.

### Neutron reflectometry

Neutron reflection measurements have been performed on the MARIA beamline^70^ operated by Jülich Centre for Neutron Science at Heinz Maier-Leibnitz Zentrum in Garching (Germany), using custom temperature-regulated liquid cells^71^. The measurements were performed using two wavelengths, 10 Å for the low-q region and 5 Å for the high-q region up to 0.25 Å^-1^, with 10% wavelength spread. Details about the instrumental setup and the technique are reported in^72^. The technique allows to access the structural properties of stratified interfaces in the cross direction. The technique is well suited to investigate the cross structuring of planar biomembranes deposited at interfaces such as in our case at the solid/liquid interface (silicon support and water)^73–75^ and it consists in sending a grazing beam to the sample and in collecting the reflected intensity as a function of q_z_, the momentum transfer normal to the interface, where q_z_ = 4λsinα/2, with α angle of beam incidence and λ incoming beam wavelength.

Reflectivity has been measured from the silicon supports and from the samples in different water solutions (H_2_O and D_2_O and eventually 4 MW, a mix of H_2_O and D_2_O with Scattering Length Density, SLD, 4*10^−6^ Å^−2^). In neutron reflectometry experiments, indeed, the systems under investigation are generally studied in different H_2_O/D_2_O mixtures to selectively enhance or lower the contrast (i.e. visibility) of different portions of the system, to determine the hydration of the layers, which contrast varies according to the solvent used. Co-refinement of multiple contrast reflectivity data leads to an overall decrease in the ambiguity of the fits and helps in obtaining sub-nanometer information about the out of plane membrane structure. An initial analysis of the neutron reflectivity results for both systems of PLPC/sitosterol 80/20 mol and PLPC/sitosterol/GluCer 60/20/20 mol on silicon was performed using a recently introduced model-free approach^41^ where no assumptions concerning the structure of the interfacial layer are made. The first step consists of estimating the overall layer thickness (D_max) by performing an IndirectFourier Transform of the reflectivity data. Subsequently a simulated annealing minimization of the discrepancy between theoretical calculated reflectivity and experimental data for multiple solvent contrasts led to an estimation of solvent volume fraction and SLD profiles near the silicon surface.

This unbiased indication from the model-free fitting approach was further explored and quantified using a more traditional model-based data fitting procedure using the program MOTOFIT^42,43^, by performing aco-refinement of the data collected in different contrast solutions: the fit of the curves was simultaneous and the values reported in the C) panels of Figs. 5 and 7 correspond to the fit parameters giving the best fit of the curves obtained in the three different contrasts, shown in panels A) of Figs. 5 and 7.

## Supplementary Information

Supplementary Information.

## References

[1] Jarsch, I. K. *et al.* Plasma membranes are Subcompartmentalized into a plethora of coexisting and diverse microdomains in Arabidopsis and *Nicotiana benthamiana*. *Plant Cell* **26** (2014). 10.1105/tpc.114.124446 PMC4036580 24714763

[2] Beck, J. G., Mathieu, D., Loudet, C., Buchoux, S. & Dufourc, E. J. Plant sterols in “rafts”: A better way to regulate membrane thermal shocks. *FASEB J.* **21** (2007). 10.1096/fj.06-7809com 17317727

[3] Xu, X. *et al.* Effect of the structure of natural sterols and sphingolipids on the formation of ordered sphingolipid/sterol domains (rafts). *J. Biol. Chem.* **276** (2001). 10.1074/jbc.M104776200 11432870

[4] Mamode Cassim, A. *et al.* Plant lipids: KEY players of plasma membrane organization and function. *Prog. Lipid Res. 73* (2019). 10.1016/j.plipres.2018.11.002 30465788

[5] Karnovsky, M. J., Kleinfeld, A. M., Hoover, R. L. & Klausner, R. D. The concept of lipid domains in membranes. *J. Cell Biol.* **94** (1982). 10.1083/jcb.94.1.1 PMC2112185 6889603

[6] Bhat, R. A., Miklis, M., Schmelzer, E., Schulze-Lefert, P. & Panstruga, R. Recruitment and interaction dynamics of plant penetration resistance components in a plasma membrane microdomain. *Proc. Natl. Acad. Sci. USA.* **102** (2005). 10.1073/pnas.0500012102 PMC549507 15703292

[7] Cacas J-L (2012). Lipids of plant membrane rafts. Prog. Lipid Res..

[8] Borner, G. H. H. *et al.* Analysis of detergent-resistant membranes in arabidopsis. Evidence for plasma membrane lipid rafts. *Plant Physiol.* **137** (2005). 10.1104/pp.104.053041 PMC548842 15618420

[9] Titapiwatanakun, B. *et al.* ABCB19/PGP19 stabilises PIN1 in membrane microdomains in Arabidopsis. *Plant J.* **57**, (2009). 10.1111/j.1365-313X.2008.03668.x 18774968

[10] Simon-Plas F, Perraki A, Bayer E, Gerbeau-Pissot P, Mongrand S (2011). An update on plant membrane rafts. Curr. Opin. Plant Biol..

[11] Guillier, C. *et al.* Direct purification of detergent-insoluble membranes from Medicago truncatula root microsomes: Comparison between floatation and sedimentation. *BMC Plant Biol.* **14** (2014). 10.1186/s12870-014-0255-x PMC4193990 25267185

[12] Yoshida, S. & Uemura, M. Protein and lipid compositions of isolated plasma membranes from orchard grass (*Dactylis glomerata* L.) and changes during cold acclimation. *Plant Physiol.* **75** (1984). 10.1104/pp.75.1.31 PMC1066829 16663596

[13] Hartmann, M. A. Plant sterols and the membrane environment. *Trends Plant Sci.* **3** (1998).

[14] Schuler, I. *et al.* Differential effects of plant sterols on water permeability and on acyl chain ordering of soybean phosphatidylcholine bilayers. *Proc. Natl. Acad. Sci. USA.* **88** (1991). 10.1073/pnas.88.16.6926 PMC52206 11607205

[15] Krajewski-Bertrand, M. A., Milon, A. & Hartmann, M. A. Deuterium-NMR investigation of plant sterol effects on soybean phosphatidylcholine acyl chain ordering. *Chem. Phys. Lipids* **63** (1992).

[16] Mora, M. P. *et al.* Optimisation of plant sterols incorporation in human keratinocyte plasma membrane and modulation of membrane fluidity. *Chem. Phys. Lipids* **101** (1999). 10.1016/s0009-3084(99)00067-5 10533266

[17] Markham, J. E., Lynch, D. V., Napier, J. A., Dunn, T. M. & Cahoon, E. B. Plant sphingolipids: Function follows form. *Curr. Opin. Plant Biol. 16* (2013). 10.1016/j.pbi.2013.02.009 23499054

[18] Msanne, J. *et al.* Glucosylceramides are critical for cell-type differentiation and organogenesis, but not for cell viability in Arabidopsis. *Plant J.* **84**, (2015). 10.1111/tpj.13000 PMC4765501 26313010

[19] Emami S (2017). Molecular dynamics simulations of ternary lipid bilayers containing plant sterol and glucosylceramide. Chem. Phys. Lipids.

[20] Gronnier J, Gerbeau-Pissot P, Germain V, Mongrand S, Simon-Plas F (2018). Divide and rule: Plant plasma membrane organization. Trends Plant Sci..

[21] Cacas J-L (2016). Revisiting plant plasma membrane lipids in tobacco: A focus on sphingolipids. Plant Physiol..

[22] Colin, L. A. & Jaillais, Y. Phospholipids across scales: lipid patterns and plant development. *Curr. Opin. Plant Biol. 53* (2020). 10.1016/j.pbi.2019.08.007 31580918

[23] Tjellstrom H, Hellgren LI, Wieslander A, Sandelius AS (2010). Lipid asymmetry in plant plasma membranes: phosphate deficiency-induced phospholipid replacement is restricted to the cytosolic leaflet. FASEB J..

[24] Grison MS (2015). Specific membrane lipid composition is important for plasmodesmata function in arabidopsis. Plant Cell.

[25] Ünnep, R. *et al.* The ultrastructure and flexibility of thylakoid membranes in leaves and isolated chloroplasts as revealed by small-angle neutron scattering. *Biochim. Biophys. Acta Bioenerg.* **1837** (2014). 10.1016/j.bbabio.2014.01.017 24508217

[26] Hodzic, A., Rappolt, M., Amenitsch, H., Laggner, P. & Pabst, G. Differential modulation of membrane structure and fluctuations by plant sterols and cholesterol. *Biophys. J.* **94** (2008). 10.1529/biophysj.107.123224 PMC2367187 18234811

[27] Marsan, M. P., Bellet-Amalric, E., Muller, I., Zaccai, G. & Milon, A. Plant sterols: A neutron diffraction study of sitosterol and stigmasterol in soybean phosphatidylcholine membranes. *Biophys. Chem.* **75** (1998). 10.1016/s0301-4622(98)00188-4 17027456

[28] Liberton, M. *et al.* Organization and flexibility of cyanobacterial thylakoid membranes examined by neutron scattering. *J. Biol. Chem.* **288** (2013). 10.1074/jbc.M112.416933 PMC3561581 23255600

[29] Nagy, G. *et al.* Kinetics of structural reorganizations in multilamellar photosynthetic membranes monitored by small-angle neutron scattering. *Eur. Phys. J. E* **36** (2013). 10.1140/epje/i2013-13069-0 23839900

[30] Oftedal L (2012). The lipopeptide toxins anabaenolysin A and B target biological membranes in a cholesterol-dependent manner. Biochim. Biophys. Acta.

[31] Mannella, C. A. Structure of the outer mitochondrial membrane analysis of x-ray diffraction from the plant membrane. *BBA Biomembr.* **645** (1981). 10.1016/0005-2736(81)90508-3 7260085

[32] Jaillais, Y. & Ott, T. The nanoscale organization of the plasma membrane and its importance in signaling: A proteolipid perspective. *Plant Physiol.* **182** (2020). 10.1104/pp.19.01349 PMC7140965 31857424

[33] Maget-Dana R (1999). The monolayer technique: A potent tool for studying the interfacial properties of antimicrobial and membrane-lytic peptides and their interactions with lipid membranes. Biochim. Biophys. Acta Biomembr..

[34] Fang K, Zou G, He P, Sheng X, Lu C (2003). Thermodynamic characterization of mixed monolayers of phosphatidylcholine and arachidic acid on different subphases. Colloids Surf. A Physicochem. Eng. Asp..

[35] Marsh D (1996). Lateral pressure in membranes. Biochim. Biophys. Acta.

[36] Gaines, G. L. *Insoluble Monolayers at Liquid-Gas Interfaces*. (Interscience Publishers, 1966).

[37] Eeman M, Deleu M, Paquot M, Thonart P, Dufrêne YF (2005). Nanoscale properties of mixed fengycin/ceramide monolayers explored using atomic force microscopy. Langmuir.

[38] Daly, T. A., Wang, M. & Regen, S. L. The origin of cholesterol’s condensing effect. *Langmuir* **27** (2011). 10.1021/la105039q PMC3107356 21319766

[39] Krause, M. R. & Regen, S. L. The structural role of cholesterol in cell membranes: From condensed bilayers to lipid rafts. *Acc. Chem. Res.* **47** (2014). 10.1021/ar500260t 25310179

[40] Varela ARP (2016). Glucosylceramide reorganizes cholesterol-containing domains in a fluid phospholipid membrane. Biophys. J..

[41] Koutsioubas, A. Model-independent recovery of interfacial structure from multi-contrast neutron reflectivity data. *J. Appl. Crystallogr.* **52** (2019). 10.1107/S1600576719003534 PMC6557181 31236091

[42] Nelson, A. Co-refinement of multiple-contrast neutron/X-ray reflectivity data using MOTOFIT. *J. Appl. Crystallogr.* **39** (2006).

[43] https://sourceforge.net/projects/motofit/.

[44] Rondelli, V. *et al.* Building a biomimetic membrane for neutron reflectivity investigation: Complexity, asymmetry and contrast. *Biophys. Chem.* **229** (2017). 10.1016/j.bpc.2017.04.011 28499578

[45] Rondelli, V. *et al.* Ganglioside GM1 forces the redistribution of cholesterol in a biomimetic membrane. *Biochim. Biophys. Acta Biomembr.* **1818** (2012). 10.1016/j.bbamem.2012.07.010 22828449

[46] Wacklin HP, Thomas RK (2007). Spontaneous formation of asymmetric lipid bilayers by adsorption of vesicles. Langmuir.

[47] Wacklin HP (2011). Composition and asymmetry in supported membranes formed by vesicle fusion. Langmuir.

[48] Rondelli, V. *et al.* Neutrons for rafts, rafts for neutrons. *Eur. Phys. J. E. Soft Matter* **36** (2013). 10.1140/epje/i2013-13073-4 23852579

[49] Léonard, A. *et al.* Location of cholesterol in DMPC membranes. A comparative study by neutron diffraction and molecular mechanics simulation. *Langmuir* **17** (2001).

[50] Klauda JB (2010). Update of the CHARMM all-atom additive force field for lipids: Validation on six lipid types. J. Phys. Chem. B.

[51] Jo S, Lim JB, Klauda JB, Im W (2009). CHARMM-GUI membrane builder for mixed bilayers and its application to yeast membranes. Biophys. J..

[52] Jo, S., Kim, T., Iyer, V. G. & Im, W. CHARMM-GUI: A web-based graphical user interface for CHARMM. *J. Comput. Chem.* **29** (2008). 10.1002/jcc.20945 18351591

[53] Jo, S., Kim, T. & Im, W. Automated builder and database of protein/membrane complexes for molecular dynamics simulations. *PLoS ONE* **2**, e880 (2007). 10.1371/journal.pone.0000880 PMC1963319 17849009

[54] Wu, E. L. *et al.* CHARMM-GUI membrane builder toward realistic biological membrane simulations. *J. Comput. Chem. 35* (2014). 10.1002/jcc.23702 PMC4165794 25130509

[55] Jorgensen, W. L., Chandrasekhar, J., Madura, J. D., Impey, R. W. & Klein, M. L. Comparison of simple potential functions for simulating liquid water. *J. Chem. Phys.* **79** (1983).

[56] Berendsen HJC, Postma JPM, van Gunsteren WF, DiNola A, Haak JR (1984). Molecular dynamics with coupling to an external bath. J. Chem. Phys..

[57] Nosé S (1984). A molecular dynamics method for simulations in the canonical ensemble. Mol. Phys..

[58] Hoover WG (1985). Canonical dynamics: Equilibrium phase-space distributions. Phys. Rev. A.

[59] Parrinello M, Rahman A (1981). Polymorphic transitions in single crystals: A new molecular dynamics method. J. Appl. Phys..

[60] Essmann U (1995). A smooth particle mesh Ewald method. J. Chem. Phys..

[61] Steinbach PJ, Brooks BR (1994). New spherical-cutoff methods for long-range forces in macromolecular simulation. J. Comput. Chem..

[62] Hess B, Bekker H, Berendsen HJC, Fraaije JGEM (1997). LINCS: A linear constraint solver for molecular simulations. J. Comput. Chem..

[63] Abraham MJ (2015). GROMACS: High performance molecular simulations through multi-level parallelism from laptops to supercomputers. SoftwareX.

[64] Humphrey W, Dalke A, Schulten KVMD (1996). Visual molecular dynamics. J. Mol. Graph..

[65] Deleu M, Crowet JM, Nasir MN, Lins L (2014). Complementary biophysical tools to investigate lipid specificity in the interaction between bioactive molecules and the plasma membrane: A review. Biochim. Biophys. Acta Biomembr..

[66] Brasseur R, Killian JA, De Kruijff B, Ruysschaert JM (1987). Conformational analysis of gramicidin-gramicidin interactions at the air/water interface suggests that gramicidin aggregates into tube-like structures similar as found in the gramicidin-induced hexagonal HII phase. Biochim. Biophys. Acta.

[67] Claereboudt, E. J. S., Eeckhaut, I., Lins, L. & Deleu, M. How different sterols contribute to saponin tolerant plasma membranes in sea cucumbers. *Sci. Rep.* **8** (2018). 10.1038/s41598-018-29223-x PMC6052070 30022094

[68] Franche, A. *et al.* Amphiphilic azobenzenes: Antibacterial activities and biophysical investigation of their interaction with bacterial membrane lipids. *Bioorg. Chem.* **94**, 103399 (2020). 10.1016/j.bioorg.2019.103399 31706683

[69] Lins L, Brasseur R (1995). The hydrophobic effect in protein folding. FASEB J..

[70] Mattauch, S. *et al.* The high-intensity reflectometer of the jülich centre for neutron science: MARIA. *J. Appl. Crystallogr.* **51**, (2018). 10.1107/S1600576718006994 PMC5988004 29896056

[71] Koutsioubas, A. Combined coarse-grained molecular dynamics and neutron reflectivity characterization of supported lipid membranes. *J. Phys. Chem. B* **120** (2016). 10.1021/acs.jpcb.6b05433 27748120

[72] Rondelli, V. *et al.* Mucin thin layers: A model for mucus-covered tissues. *Int. J. Mol. Sci.* **20** (2019). 10.3390/ijms20153712 PMC6695901 31362433

[73] Rondelli, V. *et al.* Amyloidβ Peptides in interaction with raft-mime model membranes: A neutron reflectivity insight. *Sci. Rep.* **6** (2016). 10.1038/srep20997 PMC4754687 26880066

[74] Micciulla, S., Gerelli, Y., Campbell, R. A. & Schneck, E. A versatile method for the distance-dependent structural characterization of interacting soft interfaces by neutron reflectometry. *Langmuir* **34** (2018). 10.1021/acs.langmuir.7b02971 29039954

[75] Darré, L., Iglesias-Fernandez, J., Kohlmeyer, A., Wacklin, H. & Domene, C. Molecular dynamics simulations and neutron reflectivity as an effective approach to characterize biological membranes and related macromolecular assemblies. *J. Chem. Theory Comput.* **11** (2015). 10.1021/acs.jctc.5b00635 26574275

