# Supplementary material for: Sitosterol and glucosylceramide cooperative transversal and lateral uneven distribution in plant membranes
Source: Sci Rep. 2021 Nov 3;11:21618. doi: 10.1038/s41598-021-00696-7 (PMC8566578; doi:10.1038/s41598-021-00696-7)
Supplement: Supplementary file 1 — Supplementary Information. [file 41598_2021_696_MOESM1_ESM.pdf]

Supporting Information for the manuscript

Sitosterol and glucosylceramide cooperative transversal and lateral uneven distribution in plant membranes.

V. Rondelli[1,*], A. Koutsioubas[2], J. Pršić[3], E. Deboever[4,5], J.M. Crowet[6], L. Lins[4], M. Deleu[4,*]

[1] *Department of Medical Biotechnology and Translational Medicine, Università degli Studi di Milano, Italy.*

[2] *Jülich Centre for Neutron Science at Heinz Maier-Leibnitz Zentrum, Forschungszentrum Jülich GmbH, Garching, Germany.*

[3] *Microbial Processes and Interactions Laboratory (MiPI), TERRA Research Center, Gembloux Agro-Bio Tech, Université de Liège, Gembloux, Belgique*

[4] *Laboratoire de Biophysique Moléculaire aux Interfaces, Structure Fédérative de Recherche Condorcet, TERRA Research Center, Gembloux Agro-Bio Tech, Université de Liège, Gembloux, Belgique.*

[5] *Laboratory of Natural Molecules Chemistry, Gembloux Agro-Bio Tech, University of Liège, 2, Passage des Déportés, B-5030 Gembloux, Belgium AND FytoFend S.A., rue Georges Legrand, 6, B-5032 Isnes, Belgium*[6] *Université de Reims Champagne-Ardenne, UFR Sciences Exactes et Naturelles, Reims, France*

Corresponding Authors :

Valeria Rondelli, valeria.rondelliunimi.it

Magali Deleu, magali.deleu@uliege.be

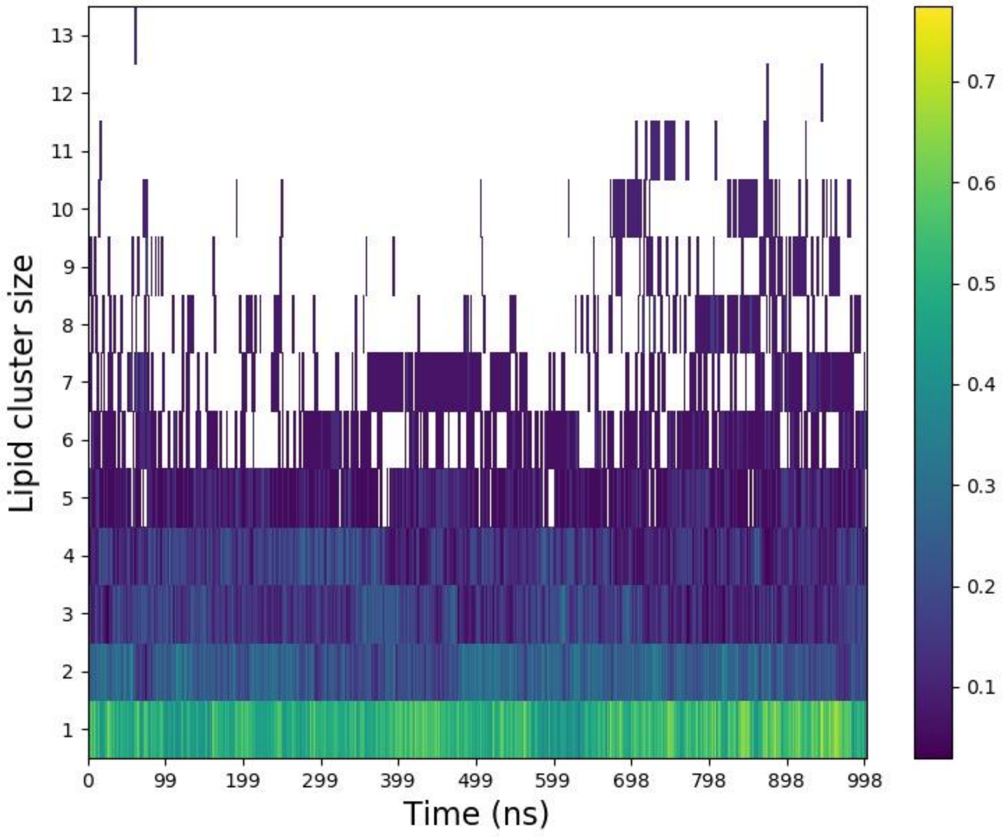

Figure S1: Proportion (color scale) of the GluCer clusters according to their size and time of the simulation. To be part of a cluster, a GluCer has to be in contact (0.35 nm cut-off) with the hydrophilic head of another GluCer from this cluster. The proportion of GluCer interacting with another GluCer (lipid cluster of 1) is around 0.5.

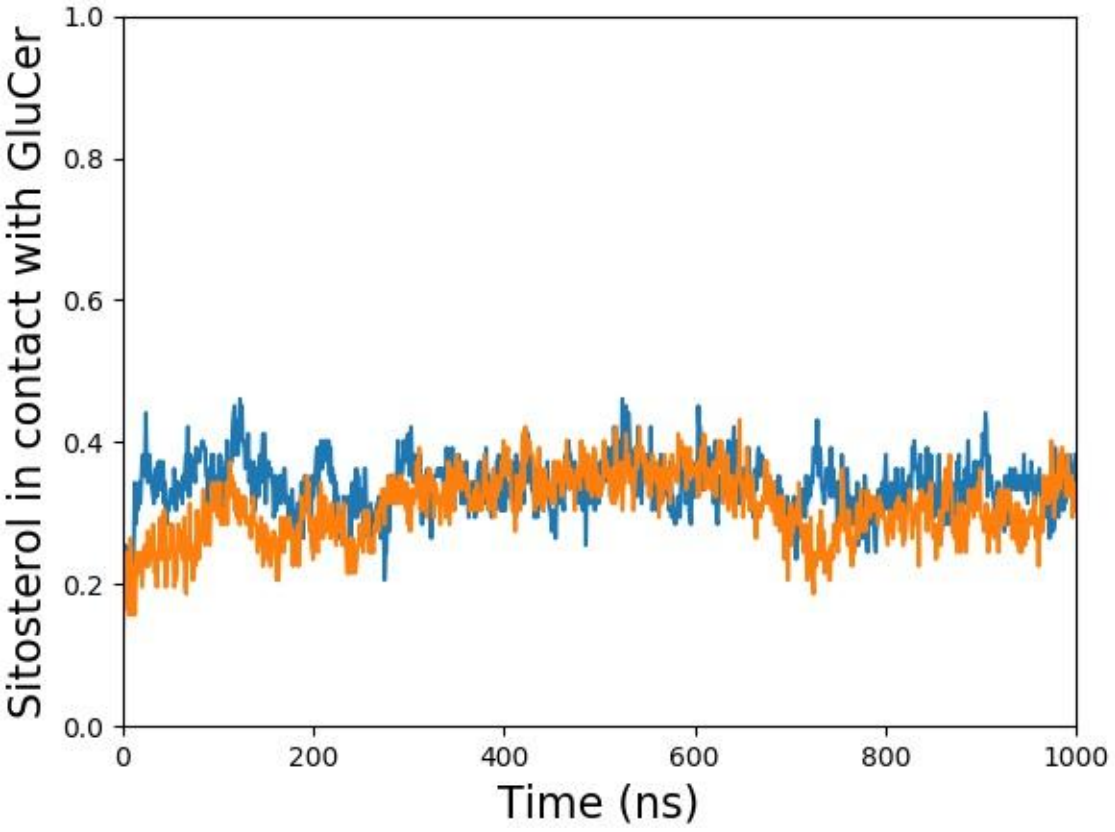

Figure S2: Proportion of Sitosterol in interaction with GluCer. Sitosterol whose hydroxyl are in contact (0.35 nm cut-off) with the hydrophilic head of GluCer are considered. The blue and orange curves correspond to the upper and lower leaflet respectively.

The ternary membrane (PLPC, sitosterol, Glucosylceramide) has been investigated several times by the deposition of freshly prepared lipid vesicles on clean silicon supports. Data have been analysed by model-free approach[1] first (Figures S1-S2) and by the software Motofit[2] after (Figures S3, S4).

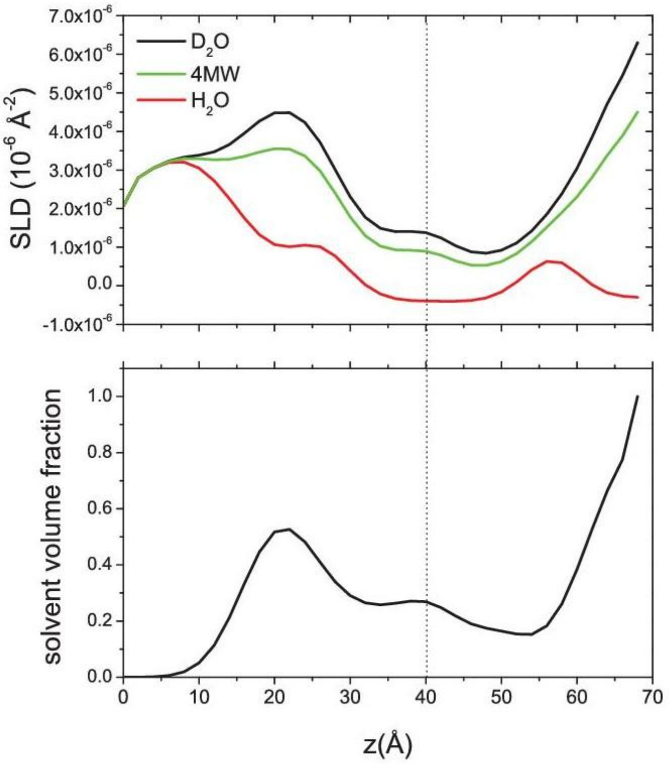

Figure S3: solvent volume fraction and sld profiles for PLPC:sitosterol:GlucCer. Dotted line represents the center between upper and lower leaflet headgroup region. (MOTOFIT analysis is reported in Figure S5 and Table S1)

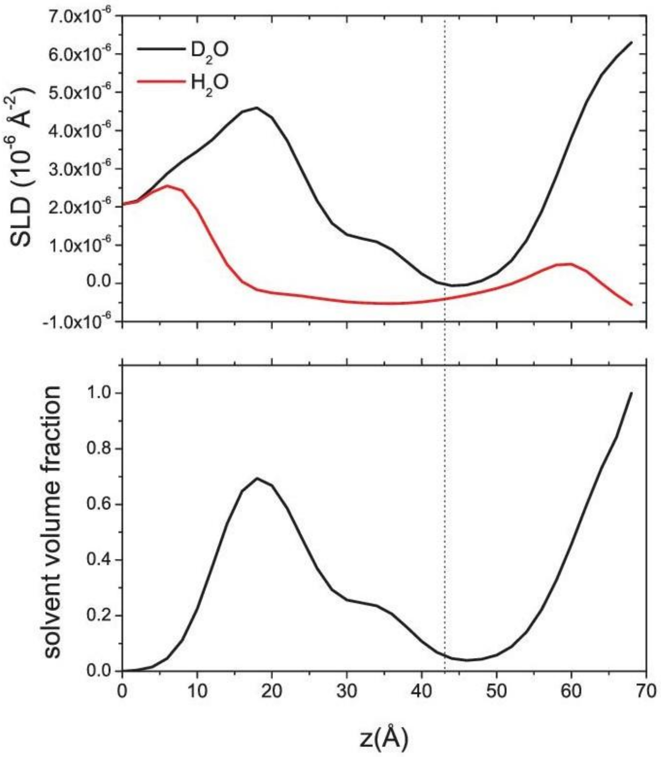

Figure S4: solvent volume fraction and sld profiles for PLPC:sitosterol:GlucCer. Dotted line represents the center between upper and lower leaflet headgroup region. (MOTOFIT analysis is reported in Figure S6 and Table S2)

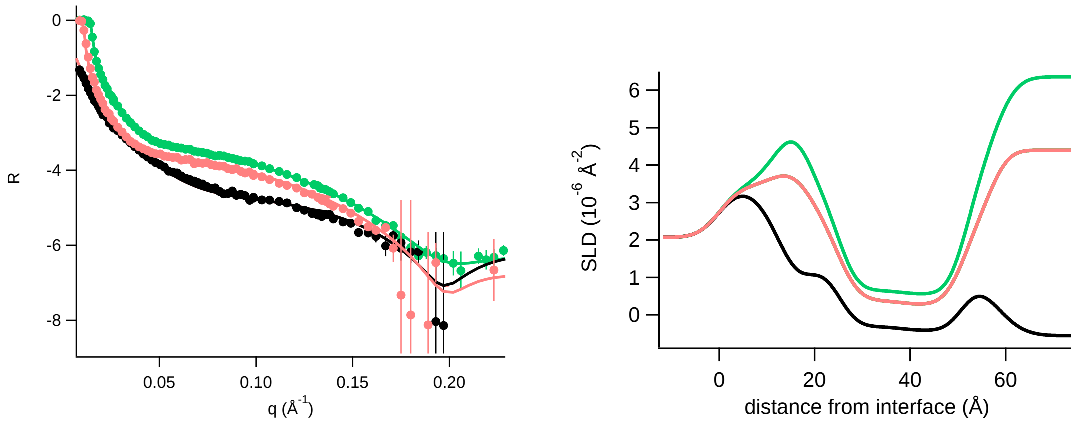

Figure S5: left: Reflectivity curves of the supported PLPC:sitosterol:GlucCer membrane investigated in TRIS buffer based on D2O (green), H2O (black) and 4MW (pink) at room temperature. Symbols are the experimental point, lines the best fits, from which the SLD profiles showed in the right panel are obtained.

Table S1. Structural parameters used to contemporary fit the curves relative to the PLPC:Sito:GlucCer membrane in the three contrast solvents. Errors on the single values have been determined from the fit quality though the χ^2 value.

	Thickness (Å)	SLD (10^{-6}Å^{-2})	Solvent penetration (%vol)
Heads in	7 ±1	1.98 ±0.04	30 ±5
Chains in	13 ±1	-0.28 ±0.03	14 ±3
Chains out	13 ±1	-0.41 ±0.03	14 ±5
Heads out	6 ±1	1.93 ±0.04	30 ±5

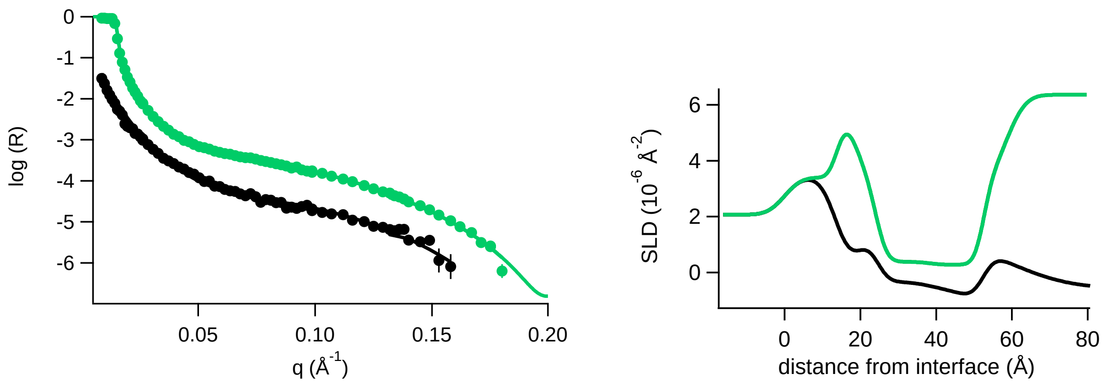

Figure S6: left: Reflectivity curves of the supported PLPC:sitosterol:GlucCer membrane investigated in D2O (green) and H2O (black) at room temperature. Symbols are the experimental point, lines the best fits from which the SLD profiles showed in the right panel have been obtained.

Table S2. Structural parameters used to contemporary fit the curves relative to the PLPC:Sito:GlucCer membrane collected in H_2O and D_2O at room temperature.

	Thickness (Å)	SLD (10^{-6}Å^{-2})	Solvent penetration (%vol)
Heads in	6 ±1	1.98 ±0.04	25 ±5
Chains in	14 ±1	-0.28 ±0.03	10 ±5
Chains out	14 ±1	-0.41 ±0.03	10 ±5
Heads out	6 ±1	1.93 ±0.04	25 ±5

References

1. A. Koutsioubas J. Appl. Cryst. 52, 538-547 (2019), DOI:10.1107/S1600576719003534
2. A. Nelson, J. Appl. Crystallogr. 39, 273–276 (2006), DOI:10.1107/S0021889806005073
